# Osmotic demyelination syndrome: Case report and literature retrospect

**DOI:** 10.1097/MD.0000000000041031

**Published:** 2024-12-20

**Authors:** Zhenzhen Yu, Chen Wang

**Affiliations:** a Department of Neurology, The Second Affiliated Hospital of Xiamen medical college, Xiamen, China; b Department of Neurology and Department of Neuroscience, The First Affiliated Hospital of Xiamen University, School of Medicine, Xiamen University, Xiamen, China.

**Keywords:** central pontine mylinolysis, etiology, extrapontine myelinolysis, osmotic demyelination syndrome, prevention

## Abstract

**Rationale:**

Osmotic demyelination syndrome (ODS) is a noninflammatory demyelinating disorder involving the pontis and other regions of the central nervous system. This article tries to enhance the understanding of ODS. Combined with clinical features, we use laboratory/imaging examination and literature review.

**Patient concerns:**

We report on 4 ODS cases and review the different etiologies, clinical manifestations, and possible pathophysiology.

**Diagnoses:**

We provide an update on the diagnosis and treatment of ODS, with a special focus on the prevention of ODS.

**Interventions:**

We corrected the hyponatremia appropriately and treated complications.

**Outcomes:**

The development of ODS is by association with a variety of clinical conditions. Also, the prognosis of ODS is heterogeneous, ranging from complete recovery to vegetative state, even death. However, there is no specific effective treatment, thus, it is more important to recognize the path mechanism of ODS and to avoid its occurrence.

**Lessons:**

It is essential to reduce the risk of suffering this potentially devastating disease through an appropriate rate of hyponatremia correction and the treatment of comorbid clinical conditions.

## 1. Introduction

Osmotic demyelination syndrome (ODS) is a rare symmetrical demyelinating disease that mainly involves the central portion of the pontis with evidence of vascular involvement. Adams first described central pontine myelinolysis (CPM) in 1959, secondary to alcoholism and malnutrition.^[1]^ Then this syndrome was shown to occur outside the pons in 1962 and referred to as extrapontine myelinolysis (EPM).^[2]^ In recent years, more and more clinical conditions have been confirmed that involve the development of ODS with various clinical manifestations because of the different brain lesions. Brain magnetic resonance imaging (MRI) is a useful tool for diagnosing ODS; however, its changes may be delayed. Thus, it often causes irreversible brain damage as a consequence of misdiagnosis if doctors are less aware of the etiology and clinical features of ODS. Hence, it is necessary to deepen our understanding of ODS. Here, we report 4 ODS cases, review the different etiologies, clinical manifestations, and possible pathophysiology, and provide an update on the diagnosis and treatment of ODS, with a special focus on the prevention of ODS.

## 2. Case reports

### 2.1. Case 1

**Figure 1.**
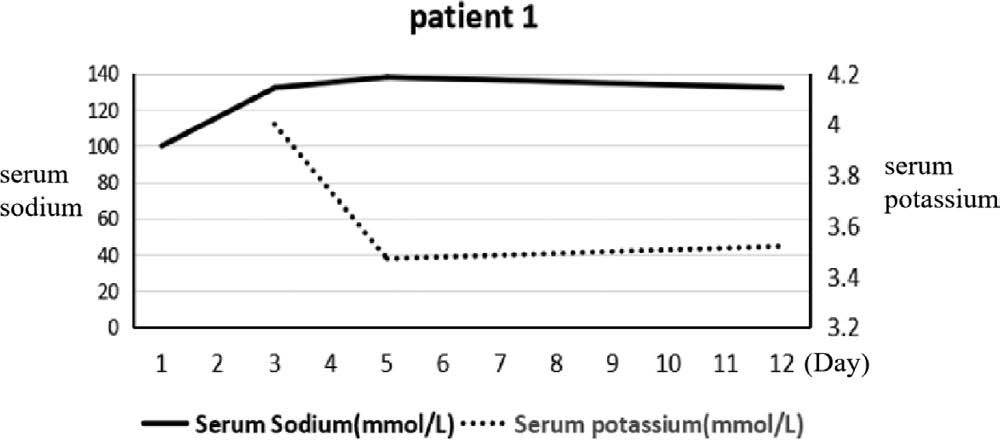
The serum sodium and potassium of patient 1. This shows a patient with hyponatremia before admission. Its correction was finished over a period of 2 days. There were different symptoms along with the concentrate changes of serum sodium.

**Figure 2.**
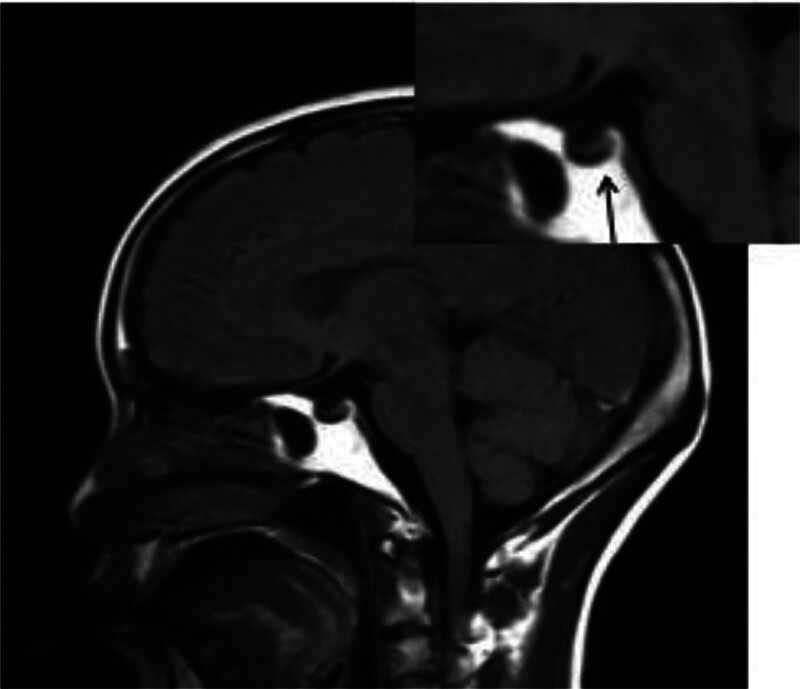
The sagittal MR images of patient 1 showing empty sella (arrow) secondary to postpartum pituitary necrosis. MR = magnetic resonance.

**Figure 3.**
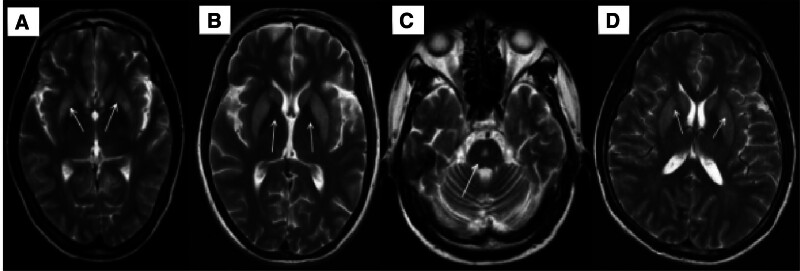
The above pictures were T2-wighted images of 4 patients. The sequence of images correspond to the case order. (A, B) and (D) show symmetrical abnormal signals in the bilateral basal ganglia. These changes are in line with the character of extra pontine myelinolysis (EPM) and (C) belongs to the change brought about by central pontine myelinolysis (CPM). CPM = central pontine myelinolysis, EPM = extra pontine myelinolysis.

A 41-year-old woman with a history of postpartum hemorrhage presented with Sheehan’s syndrome without clinical treatment 5 years prior to presentation to the emergency department with a 4-day history of cold, fever (the highest temperature was 38°C), headache, anorexia, and generalized weakness. An initial computed tomography (CT) scan of the head was normal. Blood test results were normal apart from a serum sodium level of 100 mmol/L (normal range is 135–150 mmol/L; Fig. 1). Hyponatremia correction was completed over a period of 2 days according to predetermined guidelines (The treatment of hyponatremia should take different treatment methods according to the cause, the type of hyponatremia, the onset of hyponatremia and the accompanying symptoms, so the treatment of hyponatremia should emphasize personalized, but the overall treatment measures include: eliminate the cause; correct hyponatremia; symptomatic treatment; treatment of complications).^[3]^ The patient is administered slowly while the patient is administered slowly while the blood sodium level is monitored and does not exceed 3%, calculated by the following formula: Total sodium supplement (mmol) = [142-Na + (mmol/L)]×body weight (kg) × 0.5. Total sodium chloride supplement (g) = [142-Na + (mmol/L)]×body weight (kg) × 0.03, administration slowly, monitoring blood sodium level and not exceeding 3%, according to the following formula: Total sodium supplement (mmol) = [142-Na + (mmol/L)]×body weight (kg) × 0.5. Total sodium chloride supplemental level (g) = [142-Na + (mmol/L)]×body weight (kg) × 0.03. The patient was seizure-free; however, her consciousness deteriorated and had no reaction to external sound stimulation. A physical examination revealed the level of consciousness was light coma. The limbs were used to perform automatic activities. Deep tendon reflexes were normal but plantar reflexes were bilateral extensors. The remaining neurological examination results were normal. Laboratory tests showed that her blood leukocyte count was normal; however, the percentage of neutrophils was 80.40. Hepatic and renal function tests and cerebrospinal fluid test results were unobtrusive. Thyroid function and sex steroids revealed hypopituitarism (Fig. 1). Brain MRI showed an empty sella (Fig. 2), abnormal signals in the bilateral basal ganglia, and brain swelling in the bilateral cortex (Fig. 3A). Changes in imaging confirmed the characteristics of the EPM. Finally, her first diagnosis was EPM secondary to rapid correction of hyponatremia. After 3 weeks of glucocorticoid treatment and symptomatic treatment. The patient’s level of consciousness had improved.

### 2.2. Case 2

**Figure 4.**
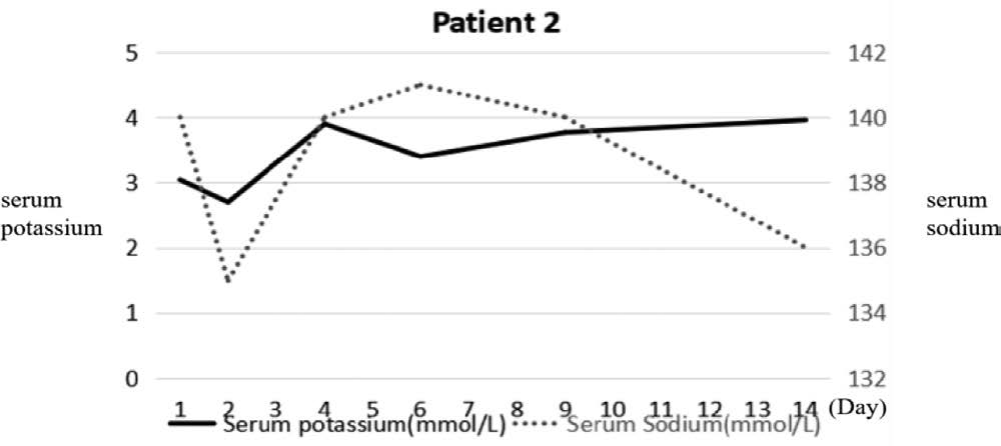
The serum sodium and potassium of patient 2 on admission. This figure reveals that this patient has hypokalemia in the course of the disease and the serum concentrates of sodium were normal.

A female patient, 43 years old, presented to our hospital with a 7-day history of fever (the highest temperature was 39°C), white water-like diarrhea (6 times a day on average), then her consciousness changed, she presented with confusion and refused to answer questions. Since the age of 23 she had received oral steroids for rheumatoid arthritis. The patient’s consciousness did not improve after treatment with antibiotics, fluid infusion, and health support. An emergency physical examination revealed sluggish grade of limb muscle strength and decreased muscle tone. Laboratory tests revealed normal cerebrospinal fluid and renal function showed mild hypokalemia (Fig. 4). Tests for rheumatoid factor and anticyclic citrullinated peptide were also positive. We considered that the fever was associated with the acute active stage of rheumatoid arthritis and the brain MRI revealed symmetrical abnormal signals in the bilateral caudate and putamen (Fig. 3B). The imaging was in accordance with the characteristics of EPM and the patient was diagnosed with EPM and electrolyte disturbances. The patient’s consciousness improved after the steroid treatment.

### 2.3. Case 3

A 51-year-old patient, male, with a 30 year history of excessive drinking presented with a history of progressive unsteady walking that manifested as lightheadedness and imbalance. These symptoms had worsened in the last 3 days, along with visual hallucinations in which the stranger always appeared, which made him agitated and upset. It was accompanied by poor focus and severe insomnia. Neurological examination revealed hypomnesia; the level and content of consciousness, grade of muscle strength, and muscle tone were normal; ataxia and Romberg’s sign were positive; and blood tests on admission showed mildly abnormal aspartate aminotransferase and gamma-glutamyl transpeptidase levels. At presentation, routine blood cell tests, hepatic renal function, and serum ammonia levels were normal. The level of blood vitamin B1 level was normal. We gradually eliminated possible causes, such as: acute alcoholism, hepatic encephalopathy, and Wernick encephalopathy. So the patient was in the condition of chronic alcoholism. Brain MRI revealed a symmetrical abnormal signal in the center of the pons (as shown in Fig. 3C). He was diagnosed with central pontine myelinolysis involving alcoholism and malnutrition. Through neurotrophic drugs, symptomatic treatment, and temperance, the patient was in remission, with improved symptoms.

### 2.4. Case 4

**Figure 5.**
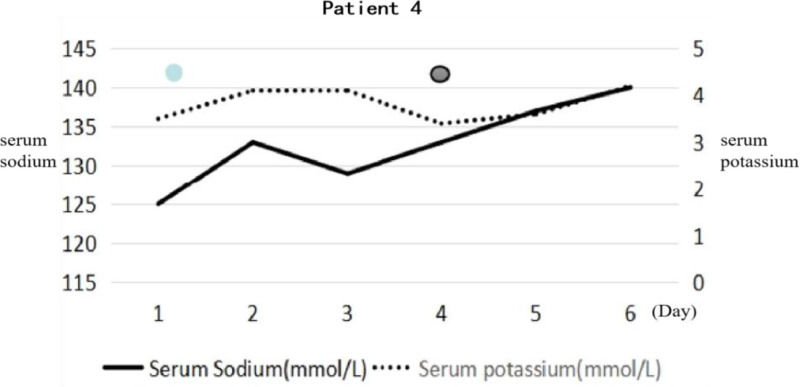
The serum sodium and potassium of patient 4. The patient was receiving parenteral nutrition after the operation. This figure revealed this patient has hyponatremia. However, with the correction for hyponatremia, the level of consciousness deteriorated rapidly. The blue spot = somnolence; the purple spot = coma with cerebral cortex failure.

A 60-year-old patient, with acute hemorrhagic necrotizing cholecystitis 12 days after a laparoscopic cholecystectomy. He presented with progressive impairment of consciousness for 4 days. It was difficult to achieve auditory comprehension and he suffered from urinary and fecal incontinence. His medical history was complicated and he had undergone a gastrectomy for a benign tumor and had treatment for a nasopharyngeal carcinoma with radiochemotherapy. Physical test: The level of consciousness was coma asyndesis, the limbs had a reaction to pain stimulation, the muscle tone of the left limb was high, and the other limbs were normal. Laboratory examination revealed hypovitaminosis, hyponatremia (Fig. 5), and moderate anemia. We considered the patient to have acute encephalopathy syndrome secondary to electrolyte disturbance and malnutrition. Through corrected electrolyte imbalance (according to the predetermined guidelines), nutritional support and symptomatic treatment, the patient’s consciousness turned in deep coma to cerebral cortex failure. The brain MRI revealed a symmetrical abnormal signal in the bilateral basal ganglia (shown in Fig. 3D). Therefore, he was diagnosed with EPM associated with multiple complications of surgery, such as malnutrition and hyponatremia, and the patient did not improve despite proactive intervention.

## 3. Discussion

Osmotic demyelination syndrome (ODS) is a rare and life-threatening demyelination syndrome involving the central nervous system that is commonly secondary to the rapid correction of severe chronic hyponatremia.^[3]^ This syndrome affects the pons and extra-pontine areas, such as the putamen, caudate, thalamus, lateral geniculate bodies, cerebral white matter, and peripheral cortex.^[4]^ Depending on the lesion area, omotic demyelination syndrome (ODS) includes CPM and extra-pontine myelinolysis (EPM), with EPM being the least common form observed in up to 20% of ODS cases.^[5]^ We report 4 cases from our hospital over the past 3 years: 1 patient with CPM and 3 patients with EPM.

ODS is thought to be associated with the rapid correction of hyponatremia, which leads to abrupt serum osmotic fluctuations. It can also occur in various clinical situations with normal serum sodium levels, such as chronic alcoholism, malnutrition, hyperglycemic state,^[6]^ hypokalemia, hypopituitarism, hypophosphatemia, hepatic failure, liver transplantation, chronic renal failure, AIDS, postoperative lithium toxicity, and malignancy.^[7,8]^ In our 4 cases, 1 patient had CPM secondary to chronic alcoholism and malnutrition, the other 3 patients were diagnosed with EPM associated with serum electrolyte disturbance. However, they had different clinical conditions such as hypopituitarism, connective tissue disease, and postsurgery.

Hyponatremia is the most common disorder of fluid and electrolyte balance in clinic. It can cause a wide range of clinical symptoms, from mild to severe and even life-threatening, and is associated with increased mortality, morbidity, and length of hospital stay for patients.^[9]^ In the first case, the female patient had hypopituitarism secondary to a postpartum hemorrhage. The patient was diagnosed with chronic hyponatremia, because hypopituitarism may result in serum electrolyte imbalance and multiple pituitary hormone deficiencies.^[10]^ Hormone deficiencies may aggravate the balance of water electrolytes when a patient is in a stressed state.^[11,12]^ Therefore, the hyponatremia in this patient worsened as an infectious condition and the seizure was considered to be induced by hyponatremia. The subsequent deterioration of consciousness appeared to be related to rapid correction of hyponatremia, which led to ODS in this case. In the fourth case, a male patient had a complicated medical history, as mentioned in a previous report. This had an impact on swallowing and nutrition absorption and put the patient in a clinical condition where malnutrition and serum electrolyte balance, gallbladder surgery, and parenteral nutrition caused the above condition to deteriorate further, especially causing acute electrolyte balance. With a correction for hyponatremia, the patient’s consciousness worsened. Therefore, the main causes of this case were the correction of hyponatremia and malnutrition. However, the pathophysiology of ODS following the rapid correction for hyponatremia is unknown, and there are some possible explanations: a change in serum osmolality can lead to impairment of the myelin sheath, whereas axons and neurons are not involved.^[13]^ The correction for hyponatremia causes downregulation of neutral amino acid transporters and impairs cellular reuptake of amino acids, making them more susceptible to damage.^[14]^

The female patient in the second case had a history of rheumatoid arthritis and received long-term oral steroid therapy. This placed her at great risk of disorders of water and electrolyte metabolism. The patient was admitted as consciousness had become an obstacle, with a history of fever and diarrhea was had been managed with intravenous fluids for 7 days at another hospital, she was referred to our hospital with encephalopathy, evaluated and it was found that hypokalemia was the main predisposing factor in this ODS case. Hypokalemia, with or without hyponatremia, has been observed in other cases of ODS.^[15]^ A study of 7 cases of central pontine myelinolysis agreed with our view.^[16]^ In another report, 89% of 74 patients with ODS had associated hypokalemia at presentation that did not normalize prior to the rapid correction of hyponatremia. The possible pathological mechanism is that the reduced endothelial cell membrane concentration of Na-K-ATPases in hypokalemia may predispose the cell to injury.^[17]^ In this case, the primary process of rheumatoid arthritis affects the blood-brain barrier integrity and easily damages the oligodendrocytes.

In the third case, chronic alcoholism was presumed to be the causative factor because the patient’s serum electrolyte levels were normal. At the same time, he had a 30 year history of excessive drinking, which is always associated with malnutrition and is characterized by a deficiency of essential nutrients. Alcoholic and malnourished individuals are generally deficient in organic osmolytes. A condition that may place them at greater risk of developing ODS.^[18]^ However, alcohol may exert toxic effects directly on pons.^[19]^ One case report revealed that beer potomania causes low-solute hyponatremia, followed by excessive diuresis with a resultant rapid increase in serum sodium concentration, which indirectly results in a change in serum osmolality and the occurrence of ODS.^[20]^

Clinically, ODS can present with a variety of symptoms and neurological features, ranging from asymptomatic to comatose, and even vegetative. This depends on the location of demyelinating lesions in the brain.^[21]^ When the pons are involved, the clinical presentation includes dysarthria and dysphagia due to the involvement of the corticobulbar tracts, flaccid quadriparesis, spasticity, corticospinal tract involvement, and oculomotor abnormalities because the fifth and sixth cranial nerves are affected. The mental status and consciousness levels may change.^[22]^ In addition, various behavioral changes, movements, and sensory disorders may be present in the EPM. These clinical features include Parkinsonism, choreoathetosis, mutism, catatonia, dysphonia, numbness of limbs, and emotional and cognitive functions are also affected.^[23]^ However, it has been reported that ODS affects the peripheral nervous system and causes bilateral and symmetrical motor demyelinating polyneuropathy in the lower and upper extremities, in association with central nervous system involvement.^[24]^ However, this hypothesis requires further confirmation.

Brain CT and MRI are useful tools for the diagnosis of ODS, particularly in patients without neurological deficits. Although demyelination lesions can occasionally be detected by brain CT as low-attenuation changes in the pons, brain MRI is a more accurate noninvasive diagnostic technique, which facilitates better anatomical characterization. Radiologically, the typical MRI findings of ODS are characterized by bilaterally symmetrical signal changes in the central pons, caudate, putamen, etc. It is unclear why the lesions are confined to these regions; however, it is postulated that the maximal admixture of gray white matter in these regions makes them more prone to changes in osmolarity. These abnormal signals present hypodensity on T1-wighted images, hyperdensity on T2-wighted images and no associated mass affecting fluid attenuation inversion recovery images. There are also atypical imaging manifestations, such as those affecting the entire central pons and bilateral swelling of the cerebral cortex. Thus, we must combine the patient’s clinical history and symptoms to diagnose ODS. Usually, MRI findings may be positive 4 weeks after the appearance of clinical manifestations; therefore, an initial negative result does not exclude ODS.^[25]^ Moreover, the severity of symptoms and prognosis are not correlated with the extent of the lesions.^[26]^ Therefore, we should avoid a premature poor prognosis based solely on the severity of imaging findings.

Once the diagnosis of ODS is established, treatment is supportive. Multiple treatment modalities include steroids, intravenous immunoglobulin, plasma exchange, thyrotrophin-releasing hormones, and administration of organic osmolytes.^[27,28]^ Although a few animal studies and case reports have demonstrated that an animal model of ODS could benefit from the reintroduction of hyponatremia,^[29]^ there is no idea whether it is an effective treatment. In addition, as ODS is not a disease in itself, but rather a complication secondary to various clinical states, more attention is focused on preventive measures. Most clinical research reminds us of minimizing the correction rate in the prevention of ODS, however, optimization of the intracellular response during the correction phase is necessary when a sufficient supply of various factors is required.^[30]^

## 4. Conclusion

The development of ODS is associated with several clinical conditions. In addition, the prognosis of ODS is heterogeneous, ranging from complete recovery to a vegetative state and even death. However, there is no specific effective treatment; thus, it is more important to recognize the mechanism of ODS and to avoid its occurrence. In short, it is essential to reduce the risk of this potentially devastating disease through appropriate correction of hyponatremia and treatment of comorbid clinical conditions.

## Acknowledgments

This study was supported by the Natural Science Fund of Fujian Province (No. 2023J011622) and Natural Science Fund of Xiamen (No. 3502Z20224ZD1259).

## Author contributions

**Conceptualization:** Chen Wang, Zhenzhen Yu.

**Data curation:** Chen Wang, Zhenzhen Yu.

**Formal analysis:** Chen Wang, Zhenzhen Yu.

**Funding acquisition:** Chen Wang, Zhenzhen Yu.

**Investigation:** Chen Wang, Zhenzhen Yu.

**Methodology:** Chen Wang, Zhenzhen Yu.

**Project administration:** Chen Wang, Zhenzhen Yu.

**Resources:** Chen Wang, Zhenzhen Yu.

**Software:** Chen Wang, Zhenzhen Yu.

**Supervision:** Chen Wang, Zhenzhen Yu.

**Validation:** Chen Wang, Zhenzhen Yu.

**Visualization:** Chen Wang, Zhenzhen Yu.

**Writing – original draft:** Chen Wang, Zhenzhen Yu.

**Writing – review & editing:** Chen Wang, Zhenzhen Yu.
